# Remedial Training of the Less-Impaired Arm in Chronic Stroke Survivors With Moderate to Severe Upper-Extremity Paresis Improves Functional Independence: A Pilot Study

**DOI:** 10.3389/fnhum.2021.645714

**Published:** 2021-03-12

**Authors:** Candice Maenza, David A. Wagstaff, Rini Varghese, Carolee Winstein, David C. Good, Robert L. Sainburg

**Affiliations:** ^1^Department of Neurology, Pennsylvania State University College of Medicine, Hershey, PA, United States; ^2^Department of Kinesiology, Pennsylvania State University, State College, PA, United States; ^3^Department of Human Development and Family Studies, Pennsylvania State University, State College, PA, United States; ^4^Department of Biokinesiology and Physical Therapy, University of Southern California, Los Angeles, CA, United States

**Keywords:** hemisphere-specific deficits, ipsilesional deficits, stroke impairment, motor deficits, stroke remediation, ipsilateral deficits

## Abstract

The ipsilesional arm of stroke patients often has functionally limiting deficits in motor control and dexterity that depend on the side of the brain that is lesioned and that increase with the severity of paretic arm impairment. However, remediation of the ipsilesional arm has yet to be integrated into the usual standard of care for upper limb rehabilitation in stroke, largely due to a lack of translational research examining the effects of ipsilesional-arm intervention. We now ask whether ipsilesional-arm training, tailored to the hemisphere-specific nature of ipsilesional-arm motor deficits in participants with moderate to severe contralesional paresis, improves ipsilesional arm performance and generalizes to improve functional independence. We assessed the effects of this intervention on ipsilesional arm unilateral performance [Jebsen–Taylor Hand Function Test (JHFT)], ipsilesional grip strength, contralesional arm impairment level [Fugl–Meyer Assessment (FM)], and functional independence [Functional independence measure (FIM)] (*N* = 13). Intervention occurred over a 3 week period for 1.5 h/session, three times each week. All sessions included virtual reality tasks that targeted the specific motor control deficits associated with either left or right hemisphere damage, followed by graded dexterity training in real-world tasks. We also exposed participants to 3 weeks of sham training to control for the non-specific effects of therapy visits and interactions. We conducted five test-sessions: two pre-tests and three post-tests. Our results indicate substantial improvements in the less-impaired arm performance, without detriment to the paretic arm that transferred to improved functional independence in all three posttests, indicating durability of training effects for at least 3 weeks. We provide evidence for establishing the basis of a rehabilitation approach that includes evaluation and remediation of the ipsilesional arm in moderately to severely impaired stroke survivors. This study was originally a crossover design; however, we were unable to complete the second arm of the study due to the COVID-19 pandemic. We report the results from the first arm of the planned design as a longitudinal study.

## Introduction

Damage to one side of the brain due to stroke often leads to upper-extremity motor impairment on the side of the body opposite to the brain lesion (Morris et al., 2008; Stinear, 2010; Mani et al., 2013). These contralesional motor impairments in the arm and hand have been extensively characterized and reflect the main focus of remedial physical rehabilitation to the upper limbs, following stroke. However, substantial evidence over the past few decades has described ipsilesional arm motor deficits that can be functionally limiting and often persist throughout the chronic stage of stroke (Wetter et al., 2005; Chestnut and Haaland, 2008; Poole et al., 2009; Schaefer et al., 2012; Metrot et al., 2013; Sainburg et al., 2016; Bustrén et al., 2017; Semrau et al., 2017; Maenza et al., 2020; Varghese and Winstein, 2020). Such deficits have been characterized through studies that use high resolution motion analysis of laboratory tasks, as well as in studies that have employed functional assessments such as the Purdue Pegboard Test, Jebsen–Taylor Hand Function Test (JHFT), and assessments of activities of daily living (Rapin et al., 1966; Winstein and Pohl, 1995; Desrosiers et al., 1996; Wetter et al., 2005; Schaefer et al., 2009b; Haaland et al., 2012; Mutha et al., 2013; Maenza et al., 2020). Many studies that have assessed kinematics of reaching movements of the ipsilesional arm have indicated that ipsilesional arm motor deficits tend to depend on the hemisphere that was damaged (Winstein and Pohl, 1995; Pohl and Winstein, 1999; Mutha et al., 2012, 2013; Schaefer et al., 2012). Specifically, left hemisphere damage (LHD) produces movement trajectory deficits, including direction errors, abnormally high movement curvatures, slow speeds and deficits in movement smoothness. Right hemisphere damage (RHD) tends to impair the ability to bring the arm to rest at an accurate and stable final position (Schaefer et al., 2009a, 2012). While ipsilesional arm motor deficits are more pronounced in patients with severe contralesional deficits, they can be substantial in patients with moderate contralesional deficits (Bustrén et al., 2017; Maenza et al., 2020; Varghese and Winstein, 2020).

Although ipsilesional arm motor deficits are less prominent than deficits in the contralesional arm, they can have a substantial impact on functional independence for stroke survivors, particularly in those with more severe contralesional arm impairment (Maenza et al., 2020). This is because stroke survivors with more severe paresis in the contralesional arm have little-to-no ability to perform functional manipulations with the hand, and thus must rely on their ipsilesional arm and hand for activities of daily living (ADL). In support of this explanation, Vega-González and Granat (2005) found that those with moderate to severe contralesional deficits tended to use their ipsilesional arm 3–6 times more than their contralesional arm (Vega-González and Granat, 2005). The tendency to rely on the ipsilesional arm for activities of daily living increases with contralesional impairment. Therefore, deficits in ipsilesional arm control and coordination can be functionally devastating for stroke survivors with more severe contralesional paresis. This is even more apparent in right-handed individuals with LHD who must learn to rely on their non-dominant arm for manipulations in unilateral activities of daily living (Rinehart et al., 2009).

There are several treatment modalities used in rehabilitation for motor deficits following a stroke, however, none of them are specifically focused on the ipsilesional arm [for a review see Hatem et al. (2016)]. The aforementioned research indicating deficits in ipsilesional arm control and coordination has not yet influenced standard of care, largely because of the lack of research that translates these findings to clinical interventions. In addition, a potential concern when targeting the ipsilesional arm in rehabilitation might be the potential deleterious effects on contralesional arm impairment, which have been suggested previously (Allred et al., 2005; Wolf et al., 2006).

Constraint induced therapy is an effective therapy technique that has been supported by substantial evidence including a major phase 3 clinical trial (Wolf et al., 2006). This treatment focuses on remediation of the paretic arm, along with constraint of the less-impaired arm, typically using a specially designed mitt over long periods of the day. The hypothetical basis of constraining the ipsilesional less-impaired arm is the concept of learned non-use, which presumes that individuals have learned not to use their paretic arm during the early stages of motor recovery, even when they may have subsequently recovered more motor function than they realize. As a result, these individuals persistently hold back the use of their paretic arm, regardless of its potential recovery. This phenomenon of non-use is not fully understood, psychologically nor neurophysiologically, notwithstanding the substantial evidence of the phenomenon in both humans (Wolf, 2007; Stewart and Cramer, 2013) and animals (Jones et al., 1989; Allred et al., 2005, 2010; Taub et al., 2006). In addition, studies in rodents have shown that training, acutely following unilateral ischemic cortical lesions, is most effective when targeted to the contralesional limb before the ipsilesional limb, and training of the less-impaired limb can interfere with subsequent paretic limb recovery (Allred et al., 2005, 2010). Thus, a potential concern when targeting the ipsilesional arm in rehabilitation might be potential deleterious effects on contralesional arm impairment. It is important to emphasize that we focused our intervention on survivors with moderate to severe contralesional paresis, who had limited to no functional use of the contralesional hand for functional grasp, release, and manipulation, and thus would be ineligible for constraint induced intervention. Constraint of the ipsilesional arm in these individuals would render them incapable of functional manipulations. In addition, while the study by Allred et al. (2005) indicated that contralesional forelimb intervention was most effective when provided prior to ipsilesional arm intervention in the acute phase of stroke, our study focused on survivors in the chronic phase.

This study directly assesses whether ipsilesional arm remedial therapy in chronic stroke survivors with significant contralesional impairment improves functional performance and independence. Although there is limited previous evidence to suggest that incorporating the ipsilesional arm into intervention can improve some outcomes (Pandian et al., 2014), the effects of specific remedial training of the ipsilesional arm on functional independence, and on contralesional impairment has not been addressed previously.

We designed a targeted remediation protocol in order to address the hemisphere-specific deficits produced by unilateral stroke. We also assessed whether ipsilesional arm training affects performance of the contralesional arm, either positively or negatively. We hypothesized that the combination of paresis in the contralesional arm, along with persistent motor deficits in the less-impaired arm, limits functional independence in chronic stroke survivors. We therefore predict that remediation focused on improving hemisphere-specific motor deficits and general dexterity in the ipsilesional arm, would improve ipsilesional arm performance and consequently will generalize to improve functional independence without detriment to the paretic arm impairment level.

## Materials and Methods

### Participants

Thirteen (five RHD, eight LHD) chronic stroke survivors with unilateral lesions were evaluated at the Penn State Milton S. Hershey Medical Center or the University of Southern California (see participant details in Table 1). Medical records and a health screening questionnaire were used to determine eligibility. Participants were screened and excluded based on a history of (1) hospitalization for substance abuse and/or psychiatric diagnosis; (2) non-stroke neurological diseases; (3) brain stem or bilateral lesions; (4) peripheral movement disorders; and (5) left-handed prior to stroke (assessed by the Edinburgh Handedness Inventory). Inclusion criteria comprised of being at least 3 months post stroke and demonstrating significant ipsilesional motor deficits (JHFT score of greater than 65 s). For reference, earlier we found that non-disabled participants take approximately 41 s to complete the JHFT with their dominant right hand (Maenza et al., 2020). To be included in the study participants also had to demonstrate moderate or severe upper-extremity contralesional motor deficits measured at Test 1 on the Upper-Extremity Fugl–Meyer (FM). While there is little consistency in classification of impairment levels in the literature (Woodbury et al., 2013; Woytowicz et al., 2017), we adopted the severe and moderate (FM score cut-off between 19 ± 2 and 47 ± 2, respectively) impairment levels defined by Woodbury et al. (2013). Given that the goal of the intervention was to improve functional independence, participants also had to demonstrate that they were not functionally independent (FIM score < 30) for one or more activities of daily living to be included in the study.

**TABLE 1:** Summary of participant information.

*N*	Age^a^	Mean (EDU)^a^	Sex	Chronicity^a,b^	Hemisphere damaged
13	60.5 + 8.1	14.31 + 2.53	12M / 1F	5.70 + 4.3	5 Right, 8 Left

*M, male; F, female. ^a^Statistics give mean + standard deviation in years. ^b^Years post stroke.*

The Pennsylvania State College of Medicine Institutional Review Board and University of Southern California Institutional Review Board approved the study protocol, and written informed consent was obtained from all participants. We had planned to conduct a crossover study; however, we were unable to complete the second arm of the study due to the COVID-19 outbreak. We re-designed our study as a longitudinal study and report the data obtained from the participants who completed the first arm of the crossover study.

### Experimental Design

**FIGURE 1:**
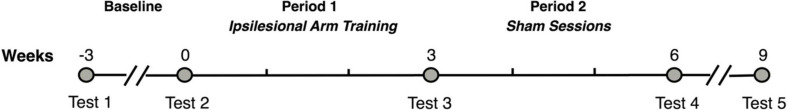
Study timeline. This figure shows the study timeline. Each participant was assessed after receiving ipsilesional arm training for 3 weeks, followed by sham sessions for another 3 weeks, and then no intervention for a 3-week retention period.

Participants completed two baseline assessments (Test 1 and Test 2) 3 weeks apart to establish stability in performance prior to ipsilesional arm training. After the second baseline assessment, participants completed 3 weeks of ipsilesional arm training (period 1) followed by another assessment (Test 3). Participants then completed 3 weeks of sham training (period 2) which was not intended to train any motor function in the less-impaired arm. Assessors were trained by a licensed occupational therapist, standardized, and blinded to whether the test was a baseline or posttest, but not blinded to the study objective. Specifically, the purpose of the sham training was to control for nonspecific effects such as the trainer’s attention, participant’s engagement, and social and motivational aspects of the sessions. Participants were assessed following the sham training (Test 4), and again 3 weeks later (Test 5) to determine if the impact of arm training performed during period 1 was maintained (see Figure 1). We predicted there would be no differences in performance between our two baseline measures, and that we would see improvement in the ipsilesional upper extremity following hemisphere-specific and general dexterity training of this arm.

#### Ipsilesional Arm Training

**FIGURE 2:**
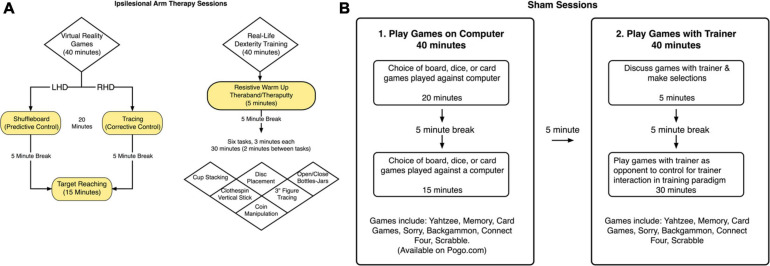
**(A)** Ipsilesional arm training paradigm. A schematized representation of the components included in the ipsilesional training program. **(B)** Sham condition. The sham condition was considered to be an attention-control condition. Sham sessions included the same frequency of lab visits and study personnel interaction, but did not include any activities that would be considered motor therapy directed toward the upper extremities.

During period 1, participants received ipsilesional arm training sessions three times a week, 1.5 h in duration (including breaks) per session, for 3 weeks. Participants first completed 40 min of hemisphere-specific virtual reality “games,” using our kinematic-virtual reality set up (Kinereach^©^), depending on which side of the brain was lesioned. These games were intended to target hemisphere-specific components of motor control that we have previously shown to be deficient in the less paretic, ipsilesional arm of stroke patients (Schaefer et al., 2007, 2009b). The arm is held above the table-top, and a cursor, representing hand position, can only be seen when the arm is maintained off the table-top. Task and movement feedback is displayed on a horizontal mirror positioned 35 centimeters above the table surface. This mirror reflects the visual stimuli presented on a horizontal, inverted, 60″ HDTV display. The first proximal interphalangeal joint of the hand reflects the position of the cursor. LHD participants engaged in a virtual shuffleboard-like game, which focused on predictive aspects of trajectory control. Participants attempted to hit a virtual puck to a target and could not make corrections once the virtual puck was touched with the virtual cursor. RHD participants engaged in tracing games, which focused on feedback mediated position control. While tracing the virtual objects they could correct themselves if they went out of the lines. They received points based on how well they stayed within the targeted path of the virtual object. After a 5-min break, all participants completed targeted reaching movements for 15 min that incorporated both trajectory control and feedback mediated position control. Participants received a score for each trial based on accuracy and speed. They were reminded of their previous score at the beginning of each session and were encouraged to try to beat it. Throughout the training, the trainer enthusiastically encouraged each participant to perform as many rapid movements as possible. In the next 40-min phase of the session, participants engaged in ipsilesional arm training that mirrored real-life activities. Specifically, they started with 5 min of preparatory mild resistive exercises of the ipsilesional arm, using theraputty and theraband, which are elastic substances designed for resistive exercises of the hand and arm, respectively. Participants were given a 5-min break before moving onto a series of six real life tasks. The trainer encouraged participants to move quickly throughout each task, and participants were given choice about the order in which they would complete each task. The trainer gave each participant 3 min to complete each task followed by a 2-min rest period. The trainer counted the number of items that the participant successfully completed in each task. This recorded count became the participant’s “target” to beat in the next session. Figure 2A displays the flow of these tasks. Although most are self-explanatory, the cup-stacking task required participants to stack 100 16-ounce disposable plastic cups as fast as they could. The rapid disks placement task used 32 two-inch diameter X 1-inch height disks. This task, which required participants to rapidly place the disks in snug-fitting rubber wells, challenges spatio-temporal accuracy and fine manipulation skills.

#### Sham Training

Following the end-of-period assessment (Test 3), participants received the sham treatment three times a week, for 1.5 h per session (including breaks), for 3 weeks. During the first part of the sham training sessions, participants played computer games for 20 min, followed by a 5-min break, and then participants played 15 more minutes of computer games (see Figure 2B). Participants had the option to choose between board, dice, or computer card games. During the second part of the sham training sessions, participants played board games (such as Battleship, Connect Four, and Yahtzee) or card games (such as “Go Fish”) for 40 min. These activities were designed to engage the ipsilesional limb nonspecifically and were assumed to not challenge the upper limb movement system to a greater extent than would be expected from usual activities of daily living.

#### Assessments

##### Jebsen–Taylor Hand Function Test

The JHFT is a clinical assessment of unilateral arm function that we used to assess ipsilesional arm performance (Beebe and Lang, 2009). The assessment simulates the coordination requirements of activities of daily living (ADL) (Jebsen et al., 1969, 1971). It includes seven timed tasks that require dexterity and arm coordination: stacking four checkers, simulated feeding, picking up small common objects (pennies, paper clips and bottle caps), writing a 24 letter sentence, moving light objects (empty weighted cans), moving heavy objects (one pound cans) and turning over 3 × 5 index cards (Jebsen et al., 1969). Because the time (seconds) to complete each of the seven tasks are summed to obtain a total score, the test assesses speed but not quality of performance. The JHFT total score has been shown to have good to excellent test-retest reliability for both dominant and nondominant hands (ICCs = 0.84–0.97) (Sığırtmaç and Öksüz, 2020). Beebe and Lang (2009) evaluated six upper limb functional tests in chronic stroke survivors, demonstrating that all six were highly correlated. Of these tests (JHFT, Grip Strength, Pinch Strength, Action Research Arm Test (ARAT), 9-Hole peg test, and the Stroke Impact Scale-Hand), the JHFT showed the greatest responsiveness and highest correlations with the other tests. In particular, the JHFT and ARAT were found to be the most comprehensive assessments of upper extremity motor function among the six tests, and highly correlated with one another (*r* = 0.87–0.95).

##### Functional independence measure

The FIM is a clinical evaluation of the level of assistance a person requires for ADLs (Granger et al., 1986). Each item is scored on a 7-point Likert scale with a score of one indicating that the person requires total assistance and a score of seven indicating that the person requires no assistance to perform the task. A score of less than five on an individual item indicates the participant cannot perform the activity without supervision and/or an aid. We used the self-care portion of the FIM, which is part of the motor subscale, and consists of eating, grooming, bathing, dressing the upper and lower body, and toileting. Participants performing these tasks could receive a maximum of 42 points. Several studies have shown the FIM motor subscale to have very high inter-rater reliability (*r* = 0.90–0.97), although exact values can vary (Ottenbacher et al., 1994; Daving et al., 2001; Glenny and Stolee, 2009). The FIM was found to have greater consistency than several other assessments used in inpatient rehabilitation and was highly correlated with the FIM Motor Subscale and the 10-item version of the Barthel Index (*r* = 0.94) (Hsueh et al., 2002).

##### Grip Strength

Ipsilesional grip strength was assessed across three trials using a hand dynamometer (Lafayette instrument, model 78010). The maximum score (kg) of the three trials was recorded. The hand dynamometer has been found to have excellent test/retest reliability (ICC 0.80 to 0.89) (Bertrand et al., 2015) and excellent intrarater and interrater reliability (ICC > 0.086–0.95) (Boissy et al., 1999) when used with individuals who have sustained a stroke. Beebe and Lang (2009) found grip strength to be highly correlated with several other upper-extremity tests at 6 months post stroke including the pinch strength test (*r* = 0.83).

### Contralesional Arm Evaluation: Upper-Extremity Fugl–Meyer

Trained staff administered the upper extremity section of the FM on the contralesional, paretic arm. The test permits researchers to differentiate proximal from distal impairment as well as assess changes in impairment over time. The motor portion of the FM has a maximum of 66 points, which indicates that the participant has no upper-extremity motor deficits. To be included in this study, participants had to demonstrate moderate or severe impairment on the FM. This study used severity cutoff values defined by Woodbury et al. (2013) in which a score between 0 and 19 + 2 indicates severe contralesional motor impairments (*N* = 8) and a score between 19 + 2 and 47 + 2 indicates moderate contralesional impairments (*N* = 5) at Test 1. It is worth noting that Woodbury’s cutoff values for each category of upper-extremity impairment do not include reflexes, which accounts for a possible six points that were included in our participant’s scores, and therefore some participants in the moderate category may actually be categorized as severe had we not included reflexes in the total score (Woodbury et al., 2013). In general, participants with severe deficits are unable to perform wrist movements, mass finger extension, or prehension FM items (Woytowicz et al., 2017). Lin et al. (2009) reported psychometric characteristics of the FM with three other tests (ARAT, UE subscale of the Stroke Rehabilitation Assessment of Movement and the Wolf Motor Function Test). Results indicated high correlations with the other tests at different time points after stroke (Spearman Correlation.82–0.96), substantial responsiveness (effect size.37–0.52), and high inter-rater reliability (ICC = 0.92–0.98).

### Statistical Design

We used Shapiro–Wilk test to assess the normality of the data obtained from our small sample. After confirming that our variances (Mauchly’s Test of Sphericity) were homogenous, we performed four separate repeated-measures analyses of variance (ANOVAs) to determine if the five test means were equal for the following four measures: the JHFT, FIM, grip strength, and FM, with “testing session” (baseline tests 1 and 2, and posttests 3, 4, 5) as the within subject factor. When a *post-hoc* analysis was warranted, we used Tukey’s test. We used JMP Pro version 15 (SAS Institute Inc.) to perform all statistical procedures with the Type I error rate set at 0.05. One participant completed Tests 1,2, and 3, but was unavailable to complete Test 4 and Test 5. We therefore report least squares means to account for the imbalance due to missing data from this participant on Test 4 and Test 5 (Cai, 2014).

## Results

### Jebsen–Taylor Hand Function Test

**FIGURE 3:**
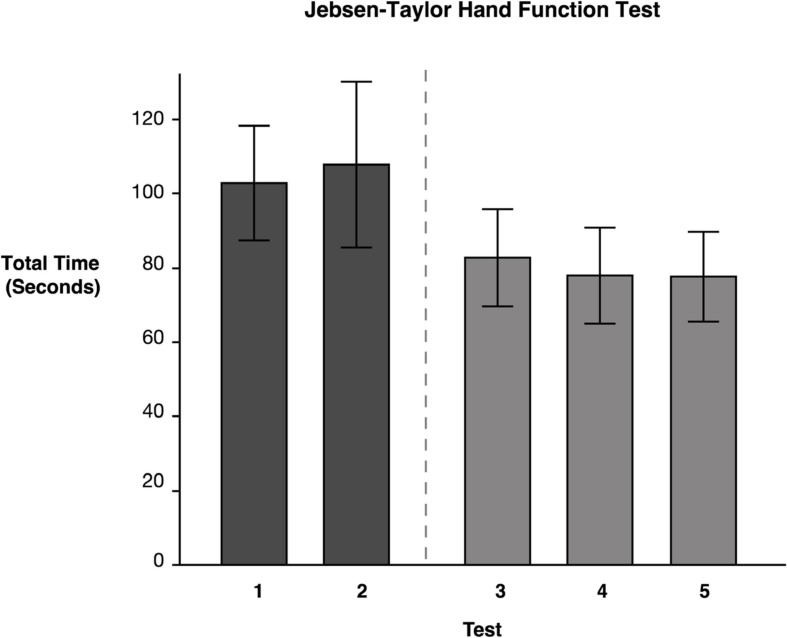
Jebsen–Taylor Hand Function Test. Least squares means and standard errors of the total time to complete the JHFT, in seconds, are shown. Participants used their ipsilesional arm. Tests 1 and 2 are the baseline measures, while tests 3–5 are posttests. All posttests showed significant improvements (*p* < 0.05) in comparison to both baseline measures.

Repeated measures ANOVA on our primary measure of ipsilesional hand function, the JHFT, indicated that there were significant mean differences between testing time points on the JHFT *F*(4, 46.05) = 6.83, *p* = 0.0002. Pairwise comparisons of the least squares means using Tukey’s HSD test (reported in Table 2) indicated that the two baseline LS means, Test 1 (*M* = 102.81) and Test 2 (*M* = 107.80), were not significantly different. In contrast, Test 3 (*M* = 82.71), administered following the last training session, showed a significant improvement, specifically a 19.21% decrease in time compared to Test 1. Test 4 (*M* = 80.23) and Test 5 (*M* = 79.98) also showed a significant improvement from Test 1 with a 21.27 and 20.72%, decrease in time, respectively; neither Test 4 nor Test 5 was significantly different from Test 3. The latter finding indicates that participants improved coordination and dexterity, and that these improvements were maintained throughout the retention period (see Figure 3).

**TABLE 2:** Tukey’s *post hoc* comparisons: Jebsen–Taylor Hand Function Test (JHFT).

T2-T1		Mean difference	Standard error mean difference	95% Confidence interval		*p*-value
Test 2	Test 1			Lower limit	Upper limit	
2	5	27.82	7.34	6.97	48.66	0.004
2	4	27.52	7.34	6.68	48.37	0.004
2	3	25.09	7.15	4.80	45.38	0.01
1	5	22.83	7.34	1.98	43.67	0.03
1	4	22.53	7.34	1.69	43.38	0.03
1	3	20.10	7.15	−0.19	40.39	0.05
2	1	4.99	7.15	−15.30	25.28	0.96
3	5	2.73	7.34	−18.11	23.58	0.10
3	4	2.44	7.34	−18.41	23.28	0.10
4	5	0.30	7.44	−20.83	21.42	1.00

### Functional Independence Measure

**FIGURE 4:**
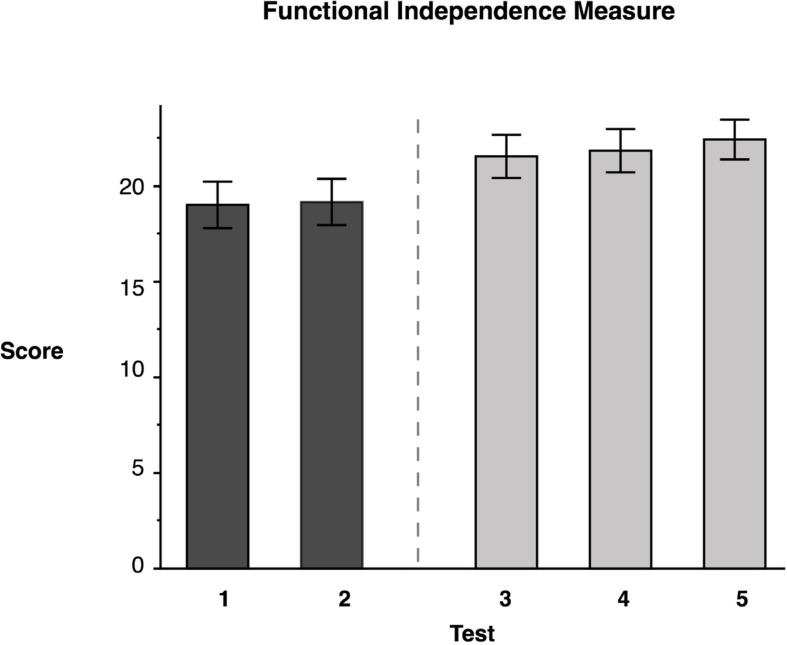
Functional Independence Measure. Least squares means and standard errors of the modified Functional Independence Measure score are shown. Tests 1 and 2 are the baseline measures, while tests 3–5 are posttests. All posttests showed significant improvements (*p* < 0.05) in comparison to both baseline measures.

Repeated measures ANOVA for our measure of functional independence, the self-care portion of the FIM, showed significant test mean differences, *F*(4, 46.02) = 34.95, *p* < 0.0001 (data reported in Table 3). As expected, Tukey’s HSD *post-hoc* comparisons indicated that the FIM LS mean scores for the two baseline measures, Test 1 (*M* = 19.0) and Test 2 (*M* = 19.15), were not significantly different from one another. In contrast, the FIM mean score immediately following training (Test 3) (*M* = 21.54) indicated that participants showed a significant 14.38% improvement from Test 1; Test 4 mean (*M* = 22.06) indicated no decline in performance during the sham period; and Test 5 (*M* = 22.64) mean observed 3 weeks later indicated that participants had retained the gains that they had made (see Figure 4). This finding indicates that targeted ipsilesional arm training had a positive, though small effect on functional independence, and that the improvement was maintained during the retention period.

**TABLE 3:** Tukey’s *post hoc* comparisons: Functional independence measure (FIM).

T2-T1		Mean difference	Standard error mean difference	95% Confidence interval		*p*-value
Test 2	Test 1			Lower limit	Upper limit	
5	1	3.64	0.41	2.49	4.79	<0.0001
5	2	3.49	0.41	2.34	4.64	<0.0001
4	1	3.06	0.41	1.91	4.21	<0.0001
4	2	2.91	0.41	1.75	4.06	<0.0001
3	1	2.54	0.39	1.42	3.66	<0.0001
3	2	2.38	0.39	1.26	3.50	<0.0001
5	3	1.10	0.41	−0.05	2.25	0.07
5	4	0.58	0.41	−0.58	1.75	0.62
4	3	0.52	0.41	−0.70	1.67	0.70
2	1	3.64	0.41	2.49	4.79	0.10

### Grip Strength

Repeated measures ANOVA showed no significant mean difference between any test for ipsilesional grip strength *F*(4, 46.08) = 0.95, *p* = 0.44. This suggests that the improvements on other tests were due to improvements in dexterity and not due to improvements in grip strength.

### Upper-Extremity Fugl–Meyer

**FIGURE 5:**
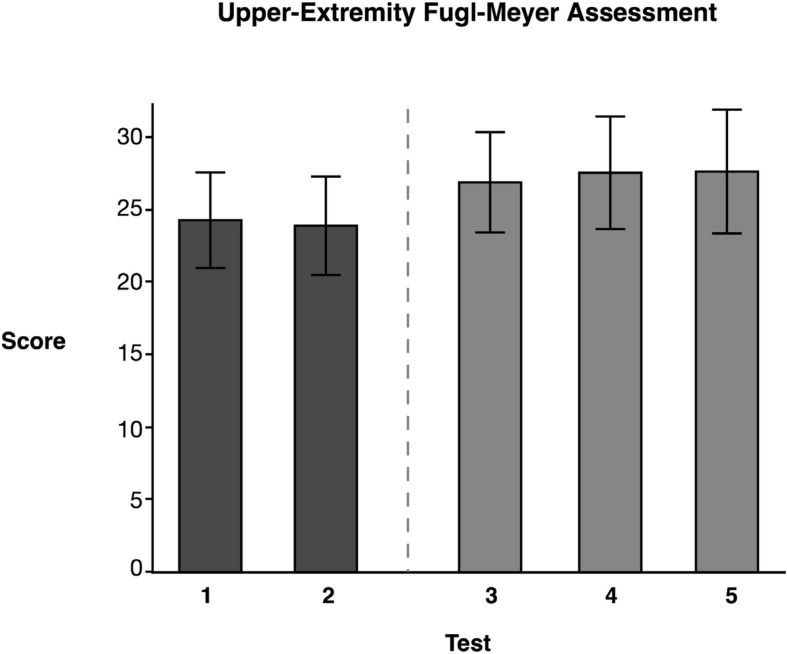
Upper-Extremity Fugl–Meyer Assessment. Least squares means and standard errors of the Upper-Extremity Fugl-Meyer Assessment are shown. Participants used their contralesional arm. Tests 1 and 2 are the baseline measures, while tests 3–5 are posttests. Not all posttests showed significant improvements (*p* < 0.05) in comparison to both baseline measures.

Repeated measures ANOVA of our measure of contralesional function, the FM Assessment, showed significant test mean differences, *F*(4, 46.02) = 4.33, *p* = 0.0054 (data reported in Table 4). Tukey’s HSD *post-hoc* comparisons indicated that the FM LS mean scores for the two baseline measures, Test 1 (*M* = 24.31) and Test 2 (*M* = 23.92) were not significantly different from one another. Moreover, the FM mean score immediately following training (Test 3), (*M* = 26.92) was not significantly different from the baseline test means. However, Test 4 (*M* = 27.20) and Test 5 (*M* = 27.28) means suggested that participants showed significant improvement compared to Test 2 (see Figure 5), but not a significant improvement from baseline 1 (see Table 4). This finding suggests that the training did not have a detrimental effect on the contralesional arm. More importantly, this finding suggests that participants experienced modest improvements (∼3 points) that were maintained during the retention period.

**TABLE 4:** Tukey’s *post hoc* comparisons: Upper-Extremity Fugl–Meyer (FM).

T2-T1		Mean difference	Standard error mean difference	95% Confidence interval		*p*-value
Test 2	Test 1			Lower limit	Upper limit	
5	2	3.36	1.15	0.08	6.63	0.04
4	2	3.27	1.15	0.00	6.55	0.05
3	2	3.00	1.12	−0.19	6.19	0.07
5	1	2.97	1.15	−0.30	6.24	0.09
4	1	2.89	1.15	−0.38	6.16	0.12
3	1	2.62	1.12	−0.57	5.80	0.15
1	2	0.38	1.12	−2.80	3.57	0.10
5	3	0.36	1.15	−2.92	3.63	0.10
4	3	0.27	1.15	−3.00	3.55	0.10
5	4	0.08	1.17	−3.23	3.40	1.00

## Discussion

In this pilot clinical intervention study, we assessed whether a rehabilitation approach focused on remediation of ipsilesional arm motor deficits in stroke survivors with moderate to severe contralesional arm paresis and with significant ipsilesional arm coordination deficits, improves functional performance and independence. This treatment was administered for a 3-week duration, three times per week, for 1.5 h per session. Each session consisted of virtual reality games, focused on hemisphere-specific deficits, followed by real-life dexterity training. We predicted that intervention-based improvements in ipsilesional arm control and coordination should improve that arm’s functional performance and should generalize to improve arm-movement-dependent aspects of functional independence. The results of this pilot study support these predictions demonstrating that ipsilesional arm training significantly improves ipsilesional arm motor performance (JHFT) and generalizes to improve functional independence. In summary, there was no significant differences in any of our measures between our two baseline assessments, which indicated that participants exhibited stability in performance on all tests prior to receiving ipsilesional arm training. Analyses of our data indicated that participants showed significant improvement with respect to ipsilateral arm motor performance (JHFT) and FIM following ipsilesional arm training and that participants maintained the improvements 3- and 6-weeks post ipsilesional arm training. Finally, the contralesional arm showed small, yet significant improvements on an impairment measure following ipsilesional arm training, supporting our prediction that training would not be detrimental to the contralesional arm.

### Remediation of the Ipsilesional Less-Impaired Arm

For the most part, physical rehabilitation of upper extremity function following stroke has understandably been focused on training movements of the contralesional arm. However, our findings provide compelling preliminary evidence that arm assessment and intervention of the less affected arm should also be incorporated into physical rehabilitation. When patients have severe contralesional paresis, the ipsilesional arm is often the primary manipulator, or even the sole manipulator. Therefore, effective performance of ADL relies upon efficient coordination of this arm and hand (Haaland et al., 2012). Our previous findings in 110 stroke survivors provides evidence that this arm often shows substantial coordination deficits that limit performance of ADL and functional independence (Maenza et al., 2020). Patients who must live with a severely paretic dominant arm, unfortunately have the most severe ipsilesional arm deficits in coordination and functional performance.

At least one previous study provided preliminary evidence that incorporating ipsilesional training into rehabilitation protocols can lead to improvements in functional independence. Pandian et al. (2014) examined whether motor intervention that was primarily focused on the ipsilesional side of the body could improve balance and functional independence in stroke survivors. In that study, the experimental group received motor rehabilitation, focusing on the nonparetic side along with conventional therapy, while the control group received only conventional therapy. The results indicated a significant effect of adding ipsilesional therapy on improvements in balance during reaching (Functional Reach Test) as well as functional independence, as measured by Barthel Index. Their study showed that incorporating ipsilesional training is important to improve whole body balance during upper limb tasks. Our study extends these findings and suggests that a therapy program that includes components specifically focused on improving ipsilesional arm motor performance shows promise for promoting improvements in functional independence, as well as a slight reduction in impairment of the paretic arm.

Our study provides the first empirical evidence albeit with a small sample that focused remediation of the ipsilesional arm may have positive effects on functional independence. However, previous studies of bilateral arm training that include the ipsilesional arm in remediation have demonstrated proven efficacy (Whitall et al., 2000; Lum et al., 2002; Luft et al., 2004; McCombe Waller and Whitall, 2004). As elegantly argued by Waller and Whitall (2008), bilateral training should always be a component of upper limb physical rehabilitation because almost all activities of daily living require bilateral actions. In addition, it is common to include the ipsilesional arm into rehabilitation, when compensatory strategies are emphasized, involving learning to perform activities unilaterally with the less-involved arm (Whitall et al., 2000). However, our current study is neither bilateral nor compensatory in nature. We instead focus on remediating hemisphere specific motor deficits in the ipsilesional arm. Neither bilateral training nor incorporation of the ipsilesional arm into compensatory strategies is likely to challenge motor performance of the ipsilesional arm to the extent of our remedial approach. In fact, Rose and Winstein (2005) studied the coordination of bilateral movements in chronic stroke survivors, and found that when requiring both arms to move together in a rapid goal-directed aiming task, the less-impaired arm adapts its speed to the slower speed of the paretic limb to preserve temporal coupling. Their finding suggests that some forms of bilateral training, while important, are unlikely to effectively challenge the motor control capacity of the ipsilesional arm. In the current study, training was focused on dexterity training and amelioration of hemisphere-specific ipsilesional arm motor control deficits. The training was graded, such that as participants improved, they were challenged to further increase the speed, accuracy, and quantity of movements. Our findings indicate that this approach improves ipsilesional arm performance, and generalizes to improved functional independence as measured by the JHFT and FIM.

### Paretic Arm Improvements

In order to monitor potential deleterious effects on contralesional paretic arm impairment, we included the upper limb portion of the FM assessment in all of our assessment sessions. We found no evidence of contralesional arm detriment as a result of ipsilesional arm training. In fact, we showed a significant, although small improvement in contralesional arm impairment, as measured by the FM assessment. It should be noted that on average, this improvement was 2.6 points at Test 3 and 3.4 points at Test 5, relative to baseline 2, and was likely too small to result in any functional gains in this cohort of moderately to severely motor impaired participants. While patients with mild to moderate motor deficits may only need a 4.25–7.25 point change in the FM score to produce meaningful changes (Page et al., 2012), patients with more severe paresis require greater changes (up to 12.4 points) in the FM score to be considered clinically meaningful (Hiragami et al., 2019).

We suggest that the small contralesional arm improvements found in our pilot study were likely due to a possible increase in participation in daily activities that might have resulted from improvements in ipsilesional arm coordination. It is also plausible that these improvements were related to interlimb transfer effects of training, a phenomenon referred to as “cross-education” (Urbin et al., 2015). Urbin et al. (2015) provided direct evidence of interlimb transfer of ipsilesional arm training, investigating whether high-intensity resistive training of the ipsilesional wrist extensors could elicit subsequent activation changes to the impaired, contralesional wrist extensors in those in the chronic stage after stroke. Their findings indicated improved ability to activate the homologous muscles in the contralesional paretic arm, following ipsilesional arm resistance training to the same muscle group. Given that training in the current study focused on coordination and not strength, as confirmed by no significant improvements in grip strength, it is unlikely to reflect the same cross-education mechanisms as reported by Urbin et al. (2015). While we do not have the ability, in the current study, to distinguish between alternative mechanisms that might account for improvements in paretic arm impairment, we suggest an activity dependent mechanism, based on potential increased participation in functional activities.

### Limitations

While this study was originally a crossover design, we were unable to complete the second limb of the study due to the COVID-19 outbreak. Because of this, the study is limited by the small cohort of participants. Though the crossover design would have yielded greater statistical power, the results from the first limb of the study, as a longitudinal design, show statistically significant and promising results. This study involved a targeted training program of the ipsilesional arm distributed over a 3-week period; it is possible that performance improvements may continue with additional therapy and therapy dose-response for this type of intervention remains untested. The results reported here were determined in patients in the chronic phase of stroke and cannot be directly generalized to the acute or subacute phases, without further research. In addition, this research was conducted on patients who reported right-handed premorbid status, and thus cannot be directly generalized to left handers.

### Conclusion

The less-impaired, ipsilesional arm of survivors of unilateral stroke is typically given little attention in rehabilitation, despite evidence of substantial, hemisphere specific, and functionally limiting motor impairments (Pohl and Winstein, 1999; Wetter et al., 2005; Schaefer et al., 2007, 2009b; Noskin et al., 2008; Poole et al., 2009; Metrot et al., 2013; Sainburg et al., 2016; Bustrén et al., 2017; Semrau et al., 2017; Maenza et al., 2020). In general, physical rehabilitation of the upper extremities following stroke, tends to focus on remediating the contralesional paretic arm, while attention to the ipsilesional arm tends to emphasize compensatory approaches, rather than remediation. However, for patients with substantial ipsilesional arm motor impairments, the effectiveness of such compensation strategies may be limited, leading to reliance on others for self-care, thus increasing the burden of care. Clinical rehabilitation has yet to realize the potential advantages of remediating ipsilesional arm motor impairments. This study provides early evidence that treating the ipsilesional arm in chronic stroke survivors can improve functional performance and independence We envision this as a first step in establishing the basis for a rehabilitation approach that focuses on remediation of each arm individually, a substantial change from current remediation protocols. The results of this study have led to a larger clinical intervention study currently ongoing, Predicting Ipsilesional Motor Deficits in Stroke with Dynamic Dominance Model (ClinicalTrials.gov identifier NCT03634397).

## Data Availability Statement

The raw data supporting the conclusions of this article will be made available by the authors, without reservation.

## Ethics Statement

The studies involving human participants were reviewed and approved by Penn State Institutional Review Board and University of Southern California Institutional Review Board. The patients/participants provided their written informed consent to participate in this study.

## Author Contributions

CM performed experiments, analyzed the data, interpreted findings, and wrote the original draft. DW and DG interpreted findings, reviewed, edited, and approved the submitted version. RV performed experiments at the USC site, reviewed, edited, and approved the submitted version. CW contributed to study design, interpreted findings, reviewed, edited, and approved the submitted version. RS conceived the research, designed the study, interpreted findings, reviewed, edited, and approved the submitted version. All authors contributed to the article and approved the submitted version.

## Conflict of Interest

CW serves as a consultant for Enspire DBS Therapy, Inc.; receives royalty payments from Human Kinetics, Inc. (for 6th edition of Motor Control and Learning), and DemosMedical Publishers (for 2nd edition of Stroke Recovery and Rehabilitation). The remaining authors declare that the research was conducted in the absence of any commercial or financial relationships that could be construed as a potential conflict of interest.
