# Supplementary material for: Remedial Training of the Less-Impaired Arm in Chronic Stroke Survivors With Moderate to Severe Upper-Extremity Paresis Improves Functional Independence: A Pilot Study
Source: Front Hum Neurosci. 2021 Mar 12;15:645714. doi: 10.3389/fnhum.2021.645714 (PMC7994265; doi:10.3389/fnhum.2021.645714)
Supplement: Supplementary file 1 [file Table_1.DOCX]

| Subject | Age^a^ | EDU^a^ | Ethnicity | Sex | Hemisphere Damaged | Chronicity^a^ | FM^b^ | JHFT Total Time (seconds)^b^ | FIM^b^ |
| --- | --- | --- | --- | --- | --- | --- | --- | --- | --- |
| 1 | 70 | 9 | Caucasian | M | Right | 9.64 | 14 | 86.6 | 15 |
| 2 | 59 | 12 | Caucasian | M | RHD | 14.88 | 20 | 80.18 | 16 |
| 3 | 60 | 16 | Caucasian | M | RHD | 3.49 | 12 | 72.17 | 12 |
| 4 | 60 | 12 | Caucasian | M | RHD | 1 | 8 | 82.58 | 16 |
| 5 | 67 | 16 | African American | M | LHD | 7.96 | 26 | 121.4 | 18 |
| 6 | 67 | 16 | Caucasian | M | LHD | 10.64 | 47 | 72.04 | 28 |
| 7 | 52 | 16 | Caucasian | M | LHD | 0.99 | 21 | 79.88 | 19 |
| 8 | 51 | 12 | Caucasian | M | LHD | 0.93 | 43 | 84.6 | 16 |
| 9 | 73 | 17 | Caucasian | M | LHD | 2.12 | 19 | 275.8 | 18 |
| 10 | 48 | 12 | Asian | M | LHD | 4.58 | 34 | 110.63 | 25 |
| 11 | 56 | 16 | Caucasian | F | LHD | 6.9 | 34 | 65.35 | 20 |
| 12 | 69 | 16 | Caucasian | M | RHD | 7.65 | 20 | 73.96 | 22 |
| 13 | 54 | 16 | Caucasian | M | LHD | 3.3 | 18 | 131.3 | 22 |

Notes: M male, F female; FM Fugl-Meyer; JHFT Jebsen-Taylor Hand Function Test; FIM Functional Independence Measure

^a^ Data given in years

^b^ Scores from baseline 1.
